# Systematic Review of the Treatment of Anosognosia for Hemiplegia in Stroke

**DOI:** 10.3390/brainsci15090906

**Published:** 2025-08-23

**Authors:** Dong Chan Kim, Junghyeon Park, Min Wook Kim

**Affiliations:** 1 Department of Rehabilitation Medicine, Eunpyeong St. Marys’ Hospital, College of Medicine, The Catholic University of Korea, Seoul, Republic of Korea, 1021, Tongil-ro, Eunpyeong-gu, Seoul 03312, Republic of Korea; dckim94@gmail.com; 2 Department of Rehabilitation Medicine, Incheon St. Marys’ Hospital, College of Medicine, The Catholic University of Korea, Seoul, Republic of Korea, 56, Dongsu-ro, Bupyeong-gu, Incheon 21431, Republic of Korea; jhp8383@naver.com

**Keywords:** anosognosia for hemiplegia, stroke, rehabilitation, self-observation

## Abstract

**Background/Objectives**: Anosognosia for hemiplegia (AHP) is a multifaceted syndrome in which stroke survivors fail to recognize motor impairments. Although AHP has significant clinical implications, rehabilitation strategies have remained fragmented and underexplored. This systematic review aimed to critically evaluate rehabilitation interventions for AHP published between 2006 and 2025, categorize intervention types, and assess clinical outcomes to inform future research and practice. **Methods**: A structured search was conducted in the PubMed and PsycINFO databases on 31 March 2025, using predefined keywords related to stroke, anosognosia, and rehabilitation. The eligible studies included randomized controlled trials, case–control studies, and case studies. Following title, abstract, and full-text screening, nine studies focusing on rehabilitation interventions for AHP were selected and analyzed. **Results**: The interventions reviewed included sensorimotor recalibration techniques, neuromodulatory approaches, error-based cognitive training, and self-observation in video replay strategies. Interventions emphasizing motor intention monitoring, error correction, and self-observation were more consistently associated with durable improvements in motor awareness than neglect-based spatial interventions were. However, many studies were limited by small sample sizes and a lack of standardized outcome measures. Assessment methodologies vary widely, highlighting the need for multidimensional theory-driven evaluation tools. **Conclusions**: Effective rehabilitation for AHP requires strategies targeting disrupted self-monitoring and agency mechanisms, rather than spatial realignment alone. The video self-observation and error-based learning paradigms show particular promise. Future research should focus on controlled trials, longitudinal tracking, and the integration of individualized, mechanism-specific rehabilitation models to optimize outcomes for stroke survivors with AHP.

## 1. Introduction

Anosognosia is classically defined as a lack of awareness or underestimation of one’s own neurological or neuropsychological impairments despite clear and observable deficits. Anosognosia, first described by Babinski in 1914, has since been documented across a range of conditions, including stroke, traumatic brain injury, and neurodegenerative disorders [1,2,3]. Rather than representing a unitary phenomenon, anosognosia is increasingly understood as a heterogeneous and multidimensional syndrome that encompasses intellectual, emergent, and anticipatory awareness and reflects disruptions across cognitive, emotional, and self-representational processes [4,5,6]. This heterogeneity is further highlighted by crucial dissociations within the syndrome that are fundamental for rehabilitation strategies. For instance, a dissociation between explicit and implicit awareness is often observed, where patients verbally deny their paralysis while their actions reveal an unconscious knowledge of their motor deficits [7]. Furthermore, the concept of emergent awareness—the recognition of a deficit only upon attempted action—is critically important, as it presents unique clinical challenges and safety risks that require distinct therapeutic approaches [8].

Recent theoretical models have emphasized that impaired self-awareness results not merely from perceptual deficits, but from broader breakdowns in self-monitoring, metacognitive processing, and integrative neural systems related to interoception, affect regulation, and agency [9,10,11]. Neuroimaging studies have suggested that anosognosia may involve disruptions in frontoparietal networks, particularly within the right hemisphere, and abnormalities in predictive coding, sensorimotor feedback, and default mode network (DMN) function [12,13,14]. Expanding on this, recent evidence increasingly conceptualizes AHP as a ‘disconnection syndrome,’ positing that the condition arises from disruptions to white matter tracts that impair communication between these critical brain networks [15]. Despite this conceptual richness, anosognosia remains inconsistently assessed in clinical practice and continues to be underrepresented in rehabilitation research [2,5].

This review focuses on anosognosia for hemiplegia (AHP), a specific manifestation wherein stroke survivors, typically following right hemisphere lesions, fail to recognize motor paralysis of the contralesional limbs. Although AHP is estimated to affect approximately 20–30% of patients with right hemisphere stroke [16], its true prevalence may be underestimated owing to its transient presentation, inconsistent clinical screening, and lack of standardized assessment tools [12,14,17]. Moreover, the AHP has rarely been addressed in formal clinical practice guidelines [2,9].

Emerging evidence underscores the substantial clinical consequences of an unrecognized AHP. Patients with AHP tend to have longer rehabilitation stays, reduced therapy participation, higher fall risk, and poorer functional outcomes [3,16,17]. Furthermore, co-occurrence with neglect, a common right hemisphere syndrome, further exacerbates disability and limits rehabilitation gains [2,6]. Notably, AHP has been shown to independently predict poorer recovery outcomes, even after adjusting for stroke severity and neglect, emphasizing the need for systematic screening and targeted intervention [9,12].

Despite their clinical importance, rehabilitation strategies targeting AHP remain fragmented and exploratory. Previous reviews have identified a limited range of interventions including caloric vestibular stimulation, mirror therapy, error-based learning, and video self-observation [2,5,16,17]. These interventions are grounded in diverse theoretical models such as sensorimotor feedback recalibration, predictive error correction, and metacognitive restructuring. However, empirical evidence remains limited because most studies involve small samples, lack randomized controlled designs, and employ heterogeneous outcome measures [11,13,14]. Moreover, interventions involving neuromodulation techniques (e.g., transcranial direct current stimulation) have been explored in more recent studies but were not extensively covered in earlier reviews, reflecting an emerging but still preliminary area of investigation.

The primary objective of this systematic review is to critically evaluate and synthesize the existing rehabilitation interventions for AHP in stroke survivors. Secondary objectives are: (1) to categorize the types of interventions based on their theoretical rationale, (2) to assess the efficacy of these interventions based on reported outcomes, and (3) to identify gaps in the current literature to propose directions for future research.

## 2. Materials and Methods

A systematic review was conducted to evaluate rehabilitation interventions targeting anosognosia for hemiplegia (AHP) following stroke. A professional research librarian assisted in the development and execution of the search strategy (Supplementary Materials Table S1). Two electronic databases, PubMed and PsycINFO, were systematically searched on 31 March 2025. Search terms included: stroke, cerebrovascular accident, anosognosia, anosognosia for hemiplegia, rehabilitation, treatment, mirror therapy, motor imagery, cognitive therapy, error-based learning, transcranial direct current stimulation (tDCS), transcranial magnetic stimulation (TMS), and virtual reality. These keywords were searched in the title and abstract fields. The search was limited to studies published since 1 January 2006.

A total of 1579 records were initially identified (PubMed = 939; PsycINFO = 640). After removing 20 duplicates, 1559 records were screened by title and abstract. Two independent reviewers assessed all articles to determine eligibility, with disagreements resolved through discussion and consensus.

Based on title and abstract review, nine articles were selected for full-text review. Of these, four were excluded: two studies were not intervention-based, one was not specific to AHP, and one was a commentary without original data. Ultimately, five studies were included from database searches. Additionally, four more eligible studies were identified through hand-searching relevant reference lists, resulting in a total of nine studies included in the final review.

**Figure 1:**
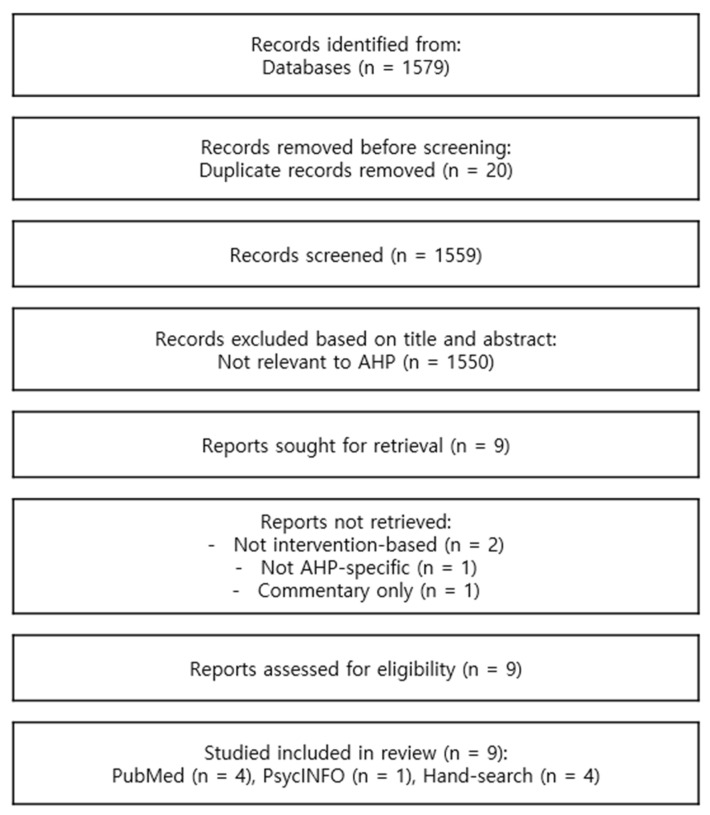
PRISMA 2020 Flow Diagram illustrating the selection process of studies for the systematic review.

The review followed PRISMA 2020 guidelines, and a completed PRISMA checklist is provided as Supplementary Materials (Table S2). A PRISMA flow diagram summarizes the study selection process (Figure 1).

The methodological quality of the nine included studies was independently assessed by two authors (D.C.K. and J.P.) using the appropriate Joanna Briggs Institute (JBI) Critical Appraisal Checklist for each study design. Any disagreements were resolved by a third author (M.W.K.) to reach a consensus. The results of the quality assessment were considered in the qualitative synthesis of the findings.

## 3. Results

Nine studies were included in this systematic review, encompassing a range of study designs, including case studies, case–control studies, and small pilot series (Table 1) [8,18,19,20,21,22,23,24,25]. The studies were conducted in Italy, France, and the UK between 2006 and 2025.

The interventions investigated varied considerably, reflecting the different theoretical models underlying the AHP. These include sensory-motor recalibration approaches (e.g., vestibular stimulation, optokinetic stimulation, and TENS), neuromodulatory techniques (e.g., tDCS), error-based cognitive training, and self-observation strategies using video replay. Most studies targeted patients with right hemisphere lesions, and most involved right-handed individuals.

The AHP assessment was heterogeneous, with tools ranging from structured clinical interviews (e.g., Bisiach Scale) to performance discrepancy measures and therapist observations. While some interventions, such as caloric vestibular stimulation [20] and video feedback [18,23,25], result in immediate or gradual improvement in motor awareness, others produce only transient or limited effects.

Overall, interventions focusing on self-monitoring, prediction error, and third-person perspective-taking appeared to be more consistently associated with awareness gains, whereas interventions originally developed for neglect (e.g., prism adaptation) demonstrated limited benefits for AHP. Methodologically, most studies were exploratory, involved small sample sizes, and lacked randomized controlled designs.

## 4. Discussion

This systematic review revealed significant heterogeneity in therapeutic outcomes, underscoring the complex and multifactorial nature of anosognosia for hemiplegia (AHP). While certain interventions resulted in measurable improvements in patients’ motor awareness, others yielded only transient effects or minimal clinical benefit. This variability indicates that AHP cannot be adequately explained as a deficit solely rooted in spatial attention or sensory neglect. Rather, it reflects broader disruptions in internal self-monitoring systems, motor intention generation, interoceptive processing, and predictive coding mechanisms that collectively sustain an individual’s awareness of action and bodily state [2,3,9]. Notably, interventions specifically targeting the recalibration of internal self-models—mechanisms by which individuals anticipate and verify their own actions—appeared to offer more promising and sustained therapeutic effects. Studies employing error-based learning paradigms [8], wherein patients confronted discrepancies between intended actions and actual motor outcomes, consistently demonstrated progressive recovery of motor awareness over time. Similarly, interventions based on video feedback and self-observation [23,25] facilitated improvements by enabling patients to adopt a third-person perspective on their own motor performance, thereby bypassing immediate, potentially impaired first-person monitoring circuits.

Conversely, interventions primarily aimed at ameliorating spatial neglect or realigning sensorimotor reference frames, such as prism adaptation or optokinetic stimulation [19], have demonstrated limited effectiveness in addressing the fundamental awareness deficits characteristic of AHP. This observation further emphasizes the conceptual distinction between AHP and neglect syndromes, highlighting the necessity for rehabilitation strategies that are specific to the underlying mechanisms.

A significant finding from this review is the increasing body of evidence supporting the efficacy of self-observation paradigms in the rehabilitation of AHP. These interventions leverage patients’ ability to address their motor deficits through externalized and temporally displaced feedback, thereby circumventing the impaired first-person monitoring systems typically associated with AHP. In a seminal study, Fotopoulou et al. demonstrated that allowing AHP patients to observe their own motor performance from a third-person, delayed perspective resulted in immediate and sustained restoration of motor awareness [18]. This finding supports theoretical models suggesting that AHP arises from a failure in first-person body schema updating, which can be bypassed by engaging higher-order metacognitive evaluation systems. Complementary results from Besharati et al. and Allum et al. indicated gradual improvements in motor awareness with repeated video feedback exposure [23,25]. These findings suggest that externalized, temporally detached feedback mechanisms can help reactivate disrupted internal self-monitoring circuits, offering a theoretically coherent and clinically actionable pathway for rehabilitation. Furthermore, individual differences, such as cognitive reserve or the emotional salience of feedback, may influence responsiveness to such interventions, underscoring the need for personalized rehabilitation approaches.

The duration of treatment varied significantly across the interventions. Caloric vestibular stimulation resulted in immediate but short-lived improvements in awareness, indicating that recalibration of multisensory integration provides only temporary stabilization of body schema representations [20]. In contrast, repetitive learning strategies [8] and self-observation interventions [23,25] exhibited more enduring and progressive improvements, supporting the notion that repeated engagement in self-monitoring and predictive coding systems is essential for sustained recovery. Furthermore, the temporal stage of stroke recovery appeared to moderately influence intervention responsiveness. Acute-phase interventions primarily aim to leverage early neuroplasticity through multisensory recalibration, whereas chronic-phase strategies focus on cognitive restructuring, prediction error processing, and reinforcement of agency attributions. These findings highlight the importance of tailoring rehabilitation strategies to the stroke recovery phase as well as the underlying cognitive targets.

Multiple studies have substantiated the notion that AHP and spatial neglect are distinct syndromes, characterized by differing neural mechanisms and responses to interventions. While treatments targeting neglect, such as prism adaptation, have been effective in ameliorating spatial biases, they exhibit minimal or no efficacy in addressing the motor awareness deficits inherent in AHP [19,24]. Conversely, interventions that focus on self-referential cognition, monitoring of motor intention, and correction of internal prediction errors have shown greater efficacy in mitigating AHP [8,18,23]. Neurocognitive models propose that AHP is associated with dysfunction in the frontoparietal networks responsible for self-monitoring and agency, whereas neglect is primarily linked to damage in the posterior parietal regions affecting external spatial representation [2,6]. These findings advocate for syndrome-specific rehabilitation strategies rather than the application of generalized attentional retraining across all right-hemisphere stroke syndromes.

The methodologies employed for assessment in studies of AHP exhibit considerable heterogeneity, reflecting the evolving conceptual frameworks within this field. While bedside tools such as the Bisiach Scale remain practical for rapid screening [19,24], they often fail to capture nuanced deficits, such as emergent or anticipatory unawareness [5]. More sophisticated approaches, such as structured discrepancy scoring [8,23] and third-person video replay assessments [18], are better suited for detecting dynamic shifts in motor self-awareness. Moro et al. distinguished between external measures (e.g., Interview for AHP, VATAm, Bisiach Scale) and internal monitoring measures (e.g., Judgement of Action Test, JAT), underscoring the dual importance of capturing both explicit and predictive awareness deficits [8]. Furthermore, validated comprehensive scales, such as the VATAm [28], UMAS [30] and MUNA [32] offer valuable frameworks that integrate self-appraisal with objective motor task performance, although these remain underutilized in intervention research. Overall, a multimodal, theoretically grounded assessment strategy is essential for accurate diagnosis, individualized treatment planning, and meaningful evaluation of rehabilitation outcomes in AHP.

Our systematic review has several limitations. First, the number of included studies was small, reflecting the paucity of intervention research in the field of AHP. Second, the included studies exhibited substantial heterogeneity in study design, intervention protocols, and outcome measures. This variability precluded quantitative synthesis, such as meta-analysis, and limited our ability to draw definitive conclusions regarding the efficacy of any specific intervention. Finally, the predominance of case reports and small case series resulted in low statistical power and limited generalizability, further restricting the strength of the evidence. These limitations highlight the need for more rigorous, large-scale research in this field.

## 5. Conclusions

This systematic review underscores the necessity for rehabilitation strategies for anosognosia for hemiplegia (AHP) to transcend traditional attentional realignment techniques, focusing instead on interventions that directly address the impaired mechanisms of self-monitoring, motor intention prediction, and metacognitive appraisal. Interventions such as error-based learning, video self-observation, and prediction-error feedback have demonstrated greater and more consistent efficacy compared to sensory-spatial recalibration methods. Furthermore, the dissociation between AHP and neglect emphasizes the need for syndrome-specific therapeutic models. Assessment strategies should progress from simple denial checklists to dynamic, multidimensional tools capable of capturing the intellectual, emergent, and anticipatory aspects of awareness. Instruments such as the VATAm [28], UMAS [30] and MUNA [32], in conjunction with structured behavioural discrepancy tasks, provide promising frameworks for future research. Moving forward, rehabilitation research should prioritize: larger, controlled trials to evaluate intervention efficacy, overcoming the persistent challenge of small sample sizes through multi-centre collaborations; longitudinal designs to monitor sustained awareness recovery; integration of theoretical models in intervention design; and tailoring interventions to the stroke recovery phase and patient-specific cognitive profiles. Recognizing AHP as a dynamic, multi-layered disorder of self-awareness opens new avenues for personalized neurorehabilitation, aiming not only to restore motor function but also to reestablish the fundamental sense of agency and body ownership in stroke survivors.

## Figures and Tables

**Table 1:** Characteristics of the included studies.

Author	Country	Study Design	Number of Cases	Brain Lesion	Handedness	AHP Assessment	Intervention	Outcome	Rationale
Fotopoulou et al., 2009 [18]	UK	Single case study	1	Right	Right-handed	Berti awareness interview [26], Feinberg awareness scale [27]	Self-observation in video replay	Observed dramatic reinstatement, instantly and permanently	judgements relying on 3rd-person and off-line self-observation
Beschin et al., 2012 [19]	Italy, UK	Comparative case study	5	Right 2; Left 3	Not reported	VATAm [28]	Optokinetic stimulation, prism adaptation, TENS	Treatment response bias, temporarily	Testing neglect-based intervention for AHP
Ronchi et al., 2013 [20]	France	Single case study	1	Left	Ambidextrous	Anosognosia score [29]	Caloric vestibular stimulation	Temporary remission of neglect, and remission of anosognosia	Vestibular input may restore awareness
Besharati et al., 2014 [21]	UK	Case–control study	8	Right	Not reported	Berti awareness interview [26], Feinberg awareness scale [27]	Negative emotional induction	Negative emotional induction improved awareness	Experimental designed study rather than a clinical study
Gandola et al., 2014 [22]	Italy	Single case study	1	Bilateral lesion on involving predominantly Right	Right-handed	Thumb-finger opposition task, Bisiach scale [29], UMAS [30]	tDCS: sham or anodal stimulation on premotor cortex	Temporary remission of AHP in the online condition with visual feedback (eyes open)	The stimulation of the premotor cortex by tDCS activates motor comparator system
Moro et al., 2015 [8]	Italy, UK	Pilot case series	4	Right: chronic stroke	Right-handed	VATAm [28], Bisiach scale [29], Moro’s interview [31], JAT [31]	Error-based training	Improved awareness	Unsuccessful action attempts with concomitant error analysis facilitate the emergent awareness
Besharati et al., 2015 [23]	UK/Italy	Experimental study	2	Right: one acute, one chronic	Right-handed	Acute: Berti awareness interview [26], Feinberg awareness scale [27] Chronic: VATAm [28], Bisiach scale [29], modified Marcel-Moro’s interview [31]	Self-observation in video replay	Improved motor awareness after video feedback	video-based, self-observation can reinstate motor awareness by providing third person and off-line feedback
Facchin et al., 2018 [24]	Italy	Single case study	1	Right	Not reported	VATAm [28], UMAS [30]	Prism adaptation	Improved neglect; AHP unchanged	Divergent improvement of neglect and anosognosia
Allum et al., 2024 [25]	UK	Qualitative case study	1	Right	Not reported	MUNA [32]	Video-based feedback during neurorehabilitation	Dramatic effects were observed, but with a subsequent recurrence	Emotion

Abbreviations: AHP = Anosognosia for Hemiplegia; JAT = Judgments of Action Test; MUNA = Motor Unawareness Assessment; tDCS = transcranial Direct Current Stimulation; TENS = Transcutaneous Electrical Nerve Stimulation; UMAS = Unawareness of Motor and somatosensory deficits After Stroke questionnaire; VATAm = Visual-Analogue Test for Anosognosia for motor impairments.

## Data Availability

No new data were created or analyzed in this study.

## Supplementary Materials

The following supporting information can be downloaded at: https://www.mdpi.com/article/10.3390/brainsci15090906/s1, Table S1: Detailed Search Strategy Used for the Systematic Review of AHP Interventions; Table S2: PRISMA 2020 Checklist.

## Abbreviations

The following abbreviations are used in this manuscript:

AHP	Anosognosia for HemiPlegia
DMN	Default Mode Network
JAT	Judgments of Action Test
MUNA	Motor Unawareness Assessment
tDCS	transcranial Direct Current Stimulation
TENS	Transcutaneous Electrical Nerve Stimulation
UMAS	Unawareness of Motor and somatosensory deficits After Stroke questionnaire
VATAm	Visual-Analogue Test for Anosognosia for motor impairments

## References

[1] Prigatano G.P. (2005). Disturbances of self-awareness and rehabilitation of patients with traumatic brain injury: A 20-year perspective. J. Head Trauma Rehabil..

[2] Orfei M.D., Caltagirone C., Spalletta G. (2009). The evaluation of anosognosia in stroke patients. Cerebrovasc. Dis..

[3] Mograbi D.C., Morris R.G. (2018). Anosognosia. Cortex.

[4] Toglia J., Kirk U. (2000). Understanding awareness deficits following brain injury. NeuroRehabilitation.

[5] Jenkinson P.M., Preston C., Ellis S.J. (2011). Unawareness after stroke: A review and practical guide to understanding, assessing, and managing anosognosia for hemiplegia. J. Clin. Exp. Neuropsychol..

[6] Langer K.G. (2009). Babinski’s anosognosia for hemiplegia in early twentieth-century French neurology. J. Hist. Neurosci..

[7] Fotopoulou A., Pernigo S., Maeda R., Rudd A., Kopelman M.A. (2010). Implicit awareness in anosognosia for hemiplegia: Unconscious interference without conscious re-representation. Brain.

[8] Moro V., Scandola M., Bulgarelli C., Avesani R., Fotopoulou A. (2015). Error-based training and emergent awareness in anosognosia for hemiplegia. Neuropsychol. Rehabil..

[9] Gainotti G. (2019). History of Anosognosia. Front. Neurol. Neurosci..

[10] Therriault J., Ng K.P., Pascoal T.A., Mathotaarachchi S., Kang M.S., Struyfs H., Shin M., Benedet A.L., Walpola I.C., Nair V. (2018). Anosognosia predicts default mode network hypometabolism and clinical progression to dementia. Neurology.

[11] Langer K.G., Bogousslavsky J. (2020). The Merging Tracks of Anosognosia and Neglect. Eur. Neurol..

[12] Barrett A.M. (2021). Spatial Neglect and Anosognosia After Right Brain Stroke. Continuum.

[13] Steward K.A., Kretzmer T. (2022). Anosognosia in moderate-to-severe traumatic brain injury: A review of prevalence, clinical correlates, and diversity considerations. Clin. Neuropsychol..

[14] Monai E., Pini L., Palacino F., Bisio M., Bernocchi F., Salvalaggio A., Corbetta M. (2023). Convergence of Visual and Motor Awareness in Human Parietal Cortex. Ann. Neurol..

[15] Pacella V., Foulon C., Jenkinson P.M., Scandola M., Bertagnoli S., Avesani R., Fotopoulou A., Moro V., de Schotten M.T. (2019). Anosognosia for hemiplegia as a tripartite disconnection syndrome. eLife.

[16] Kortte K.B., Hillis A.E. (2011). Recent trends in rehabilitation interventions for visual neglect and anosognosia for hemiplegia following right hemisphere stroke. Future Neurol..

[17] Byrd E.M., Jablonski R.J., Vance D.E. (2020). Understanding Anosognosia for Hemiplegia After Stroke. Rehabil. Nurs..

[18] Fotopoulou A., Rudd A., Holmes P., Kopelman M. (2009). Self-observation reinstates motor awareness in anosognosia for hemiplegia. Neuropsychologia.

[19] Beschin N., Cocchini G., Allen R., Della Sala S. (2012). Anosognosia and neglect respond differently to the same treatments. Neuropsychol. Rehabil..

[20] Ronchi R., Rode G., Cotton F., Farnè A., Rossetti Y., Jacquin-Courtois S. (2013). Remission of anosognosia for right hemiplegia and neglect after caloric vestibular stimulation. Restor. Neurol. Neurosci..

[21] Besharati S., Forkel S.J., Kopelman M., Solms M., Jenkinson P.M., Fotopoulou A. (2014). The affective modulation of motor awareness in anosognosia for hemiplegia: Behavioural and lesion evidence. Cortex.

[22] Gandola M., Sedda A., Manera M., Pingue V., Salvato G., Spitoni G.F., Pistarini C., Giorgi I., Pizzamiglio L., Bottini G. (2014). Selective improvement of anosognosia for hemiplegia during transcranial direct current stimulation: A case report. Cortex.

[23] Besharati S., Kopelman M., Avesani R., Moro V., Fotopoulou A.K. (2015). Another perspective on anosognosia: Self-observation in video replay improves motor awareness. Neuropsychol. Rehabil..

[24] Facchin A., Beschin N. (2018). Different impact of prism adaptation rehabilitation in spatial neglect and anosognosia for hemiplegia. Ann. Phys. Rehabil. Med..

[25] Allum J., Whittaker M., Green H. (2024). Knowing and not knowing: Practical reflections on video based feedback as part of neuro-rehabilitation in a case of persistent anosognosia for hemiplegia. Neurocase.

[26] Berti A., Ladavas E., Della Corte M. (1996). Anosognosia for hemiplegia, neglect dyslexia, and drawing neglect: Clinical findings and theoretical considerations. J. Int. Neuropsychol. Soc..

[27] Feinberg T.E., Roane D.M., Ali J. (2000). Illusory limb movements in anosognosia for hemiplegia. J. Neurol. Neurosurg. Psychiatry.

[28] Della Sala S., Cocchini G., Beschin N., Cameron A. (2009). VATA-m: Visual-Analogue Test assessing Anosognosia for motor impairment. Clin. Neuropsychol..

[29] Bisiach E., Vallar G., Perani D., Papagno C., Berti A. (1986). Unawareness of disease following lesions of the right hemisphere: Anosognosia for hemiplegia and anosognosia for hemianopia. Neuropsychologia.

[30] Nimmo-Smith I., Marcel A.J., Tegnér R. (2005). A diagnostic test of unawareness of bilateral motor task abilities in anosognosia for hemiplegia. J. Neurol. Neurosurg. Psychiatry.

[31] Moro V., Pernigo S., Zapparoli P., Cordioli Z., Aglioti S.M. (2011). Phenomenology and neural correlates of implicit and emergent motor awareness in patients with anosognosia for hemiplegia. Behav. Brain Res..

[32] Moro V., Besharati S., Scandola M., Bertagnoli S., Gobbetto V., Ponzo S., Bulgarelli C., Fotopoulou A., Jenkinson P.M. (2021). The Motor Unawareness Assessment (MUNA): A new tool for the assessment of anosognosia for hemiplegia. J. Clin. Exp. Neuropsychol..

