# Supplementary material for: Systematic Review of the Treatment of Anosognosia for Hemiplegia in Stroke
Source: Brain Sci. 2025 Aug 23;15(9):906. doi: 10.3390/brainsci15090906 (PMC12467583; doi:10.3390/brainsci15090906)
Supplement: Supplementary file 1 [file brainsci-15-00906-s001.zip › Table S2 (Supplementary Materials).pdf]

Table S2. PRISMA 2020 Checklist

Section and Topic	Item #	Checklist item	Location where item is reported
TITLE	1	Identify the report as a systematic review.	Title page
ABSTRACT	2	See the PRISMA 2020 for Abstracts checklist.	Abstract
INTRODUCTION	3	Describe the rationale for the review in the context of existing knowledge.	Introduction
INTRODUCTION	4	Provide an explicit statement of the objective(s) or question(s) the review addresses.	Introduction
METHODS	5	Specify the inclusion and exclusion criteria for the review and how studies were grouped for the syntheses.	Methods
METHODS	6	Specify all databases, registers, websites, organisations, reference lists and other sources searched or consulted to identify studies. Specify the	Methods

		date when each source was last searched or consulted.	
METHODS	7	Present the full search strategies for all databases, registers and websites, including any filters and limits used.	Supplementary Material
METHODS	8	Specify the methods used to decide whether a study met the inclusion criteria of the review.	Methods
METHODS	9	Specify the methods used to collect data from reports, including how many reviewers collected data from each report, whether they worked independently, and any processes for obtaining or confirming data from study investigators.	Methods
METHODS	10	List and define all outcomes for which data were sought. Specify whether all results that were compatible with each outcome domain in each study were sought	Methods

		(e.g. for all measures, time points, analyses), and if not, the methods used to decide which results to collect.	
METHODS	11	Specify the methods used to assess risk of bias in the included studies.	Not conducted
METHODS	12	Specify for each outcome the effect measure(s) (e.g. risk ratio, mean difference) used in the synthesis or presentation of results.	Narrative synthesis
METHODS	13	Describe the methods of handling data and combining results of studies, if done, including measures of consistency for each meta-analysis.	Narrative synthesis
METHODS	14	Describe any methods used to explore possible causes of heterogeneity among study results (e.g. subgroup analysis, meta-regression).	Narrative Discussion
METHODS	15	Describe any sensitivity analyses conducted to assess robustness of the	Not applicable

		synthesized results.	
RESULTS	16	Describe the results of the search and selection process, from the number of records identified in the search to the number of studies included in the review, ideally using a flow diagram.	Results and Figure 1
RESULTS	17	Cite each included study and present its characteristics.	Results and Table 1
RESULTS	18	Present assessments of risk of bias for each included study.	Not conducted
RESULTS	19	For all outcomes, present, for each study: (a) simple summary data for each intervention group (b) effect estimates and confidence intervals, ideally with a forest plot.	Table 1
RESULTS	20	Present results of all investigations of possible causes of heterogeneity among study results.	Discussion
RESULTS	21	Present results of all sensitivity analyses conducted to assess the robustness of the synthesized	Not applicable

		results.	
DISCUSSION	22	Provide a general interpretation of the results in the context of other evidence.	Discussion
DISCUSSION	23	Discuss any limitations of the evidence included in the review.	Discussion
DISCUSSION	24	Discuss any limitations of the review processes used.	Discussion
DISCUSSION	25	Discuss implications of the results for practice, policy, and future research.	Conclusion
OTHER INFORMATION	26	Provide registration information for the review, including register name and registration number, or state that the review was not registered.	Not registered
OTHER INFORMATION	27	Indicate where the review protocol can be accessed, or state that a protocol was not prepared.	No protocol prepared
OTHER INFORMATION	28	Describe and explain any amendments to information provided at registration or in the protocol.	Not applicable

OTHER INFORMATION	29		Describe sources of financial or non-financial support for the review, and the role of the funders or sponsors in the review.	Acknowledgements
OTHER INFORMATION	30		Declare any competing interests of review authors.	Competing Interests
OTHER INFORMATION	31		Report which of the following are publicly available and where they can be found: template data collection forms; data extracted from included studies; data used for all analyses; analytic code; any other materials used in the review.	Available on request
